# *Candida auris* Urinary Tract Infections and Possible Treatment

**DOI:** 10.3390/antibiotics9120898

**Published:** 2020-12-12

**Authors:** Nicole Griffith, Larry Danziger

**Affiliations:** 1 Department of Pharmacy Practice (m/c 833), University of Illinois at Chicago College of Pharmacy, Chicago, IL 60612, USA; ncg13@uic.edu; 2 Department of Medicine, Division of Infectious Disease, University of Illinois at Chicago College of Medicine, UIC College of Pharmacy, Suite 164 833 S. Wood Street, Chicago, IL 60612, USA

**Keywords:** *Candida auris*, urinary tract infection, antifungal resistance

## Abstract

*Candida auris* is a globally emerging pathogen that has been identified in urinary tract infections (UTIs) worldwide. The novel pathogen is characterized by common misidentification, difficult eradication, and multidrug resistance. To date, there is a paucity of data to guide the optimal management of *C. auris* UTIs. This review provides an overview of *C. auris* as an etiologic agent of UTIs, a comprehensive review of published data on *C. auris* UTIs, and a proposed treatment algorithm based on patient clinical status, the presence or absence of clinical infection, comorbidities, infection, and therapy history. Echinocandin and liposomal amphotericin B are recommended as first-line agents for most patients with *C. auris* isolated in the urine, with a focus on infection control measures and appropriate follow-up criteria. A variety of combination therapies, flucytosine, and amphotericin B bladder irrigations are offered as potential alternatives in the event of infection persistence or recurrence. The treatment approach centers on the aggressive treatment of *C. auris* in most patients, with the goal of preventing subsequent invasive spread, multi-drug resistance, and ultimate mortality. Published literature on *C. auris* urinary isolation and treatment is imperative for the future evolution of evidence-based treatment recommendations for this unique pathogen of concern.

## 1. Introduction

Infections of the urinary tract are one of the most frequently diagnosed infections in both community and hospital settings [1,2,3]. The etiologic agents of these infections may either be fungi or bacteria [3]. There is increasing evidence that the percentage of urinary tract infections (UTIs) caused by gram negative bacteria has been decreasing in recent years, while rates of UTIs caused by fungi have increased over the same time period [1,3,4]. Although *Candida albicans* is the most reported candida species found in urinary tract infections, other species such as *glabrata, tropicalis, parapsilosis, lusitanae*, and *guilliermondii* have also been identified as infecting pathogens [5,6]. In recent years, *Candida auris* has been identified as the etiologic agent in UTIs throughout the world [5,6,7].

*Candida auris* is a globally emerging organism characterized by virulence and pathogenicity, with mortality rates as high as 60% [7]. Treatment recommendations in the presence of clinical infection with *C. auris* are not thoroughly delineated, creating a challenge for treating clinicians [7]. *C. auris* possess four characteristics that make it an infecting pathogen of concern: it is commonly misidentified by available identification mechanisms, it is persistent, it frequently causes recurrent infections, and it is often resistant to available antifungals [8,9].

The purpose of this review is to discuss these characteristics, their impact on treatment considerations, and detail preferred treatment algorithms for *C. auris* UTIs.

## 2. Epidemiology/Pathophysiology/Risk Factors

*Candida auris* was first identified in Japan in 2009 after being isolated from external ear canal discharge from a 70-year old, female inpatient [10]. The pathogen was then retrospectively identified in a fungemia patient dating back to 1996 [11]. Multiple strains, or clades, of *C. auris* have now been identified across 41 countries, with presumed wider, unknown spread due to lack of reporting [7]. The first identified case of *C. auris* in the United States was documented in 2015, with a total of 1302 confirmed cases reported as of September 2020 [7]. The first published report of *C. auris* in the urine dates to 2010–2014, when urinary isolates collected from a population in India originally identified by the Vitek 2 system as *Candida haemulonii* or *Candida famata* were later identified as *C. auris* utilizing matrix-assisted laser desorption ionization–time of flight mass spectrometry (MALDI-TOF MS) [12].

*Candida auris* is frequently undetected or misidentified by traditional identification methods, as is represented by the misidentification of the first *C. auris* urinary isolates detailed above [8,10,11,12,13]. Although methods and software that can properly detect the species are now available, these updated methods may not be utilized or available within all healthcare systems [7]. The inability of common methods to accurately identify *C. auris* can lead to undiagnosed infections as well as environmental spread of infection, contributing to its status as a pathogen of global concern.

*Candida auris* is also persistent in nature, both as an environmental pathogen and an infecting agent [14,15,16,17]. Its significant ability to persist on surfaces is not completely understood but is largely attributed to biofilm formation that can survive common cleaning detergents and processes, increasing the adherence of *C. auris* to surfaces such as medical instruments, with reports of the pathogen surviving up to 14 days on medical devices [14,15,16]. *C. auris* is known to persistently colonize or infect patients for prolonged periods of time, with one report indicating an average of 49.5 days between the first and last urine culture positive for *C. auris* [17]. This persistence in the environment allows for significant spread among at risk patients in an institution [7]. Persistent infection or colonization even after presumptively adequate therapy in an individual patient can lead to inadequate control, invasive infections, and the development of resistance.

Compounding the intrinsically virulent and pathogenic characteristics of *C. auris* are its resistance trends. Based on previous reports, approximately 40% of *C. auris* isolates are resistant to ≥ 2 drug classes, and 10% are predicted to be resistant to all three classes of available antifungal drugs, with several case reports of pan-resistant *C. auris* having been reported around the world [7,18]. *C. auris* is commonly resistant to fluconazole, with approximately 90% of isolates tested in the United States possessing resistance to the drug [7]. Fluconazole resistance mechanisms are not completely understood, but are known to include EGR11 mutations and duplications, as well as efflux pumps [18,19]. *C. auris* resistance to the rest of the triazole drug class is less well defined, although some mechanisms that confer fluconazole resistance are assumed to also confer general azole resistance, and may vary based on geography and clade [7,18,19]. It is recommended to utilize fluconazole resistance as an indicator of triazole resistance, but some fluconazole-resistant strains may be susceptible to other triazoles [7]. Resistance to amphotericin B has also been identified, with an estimated 30% rate of resistance among identified isolates [7]. Current hypothesized mechanisms of polyene resistance include nonsynonymous mutations in transcription factors and non-mutation-based resistance at the transcription level [20]. Echinocandin resistance is less frequently observed, however it is seen, due to mutations in the FKS1 gene [8,20,21]. Flucytosine resistance is uncommon but can occur due to FUR1 gene mutations, and based on experience with other Candida species is likely to develop during the course of therapy, especially with flucytosine monotherapy [22]. The potential for multi-drug resistant *C. auris* isolates make antifungal treatment decisions for this pathogen incredibly difficult.

Although *C. auris* is a novel Candida species in many respects, the patient-specific risk factors for infection with *C. auris* are similar to those seen with other Candida infections [7,9]. While *C. auris* does not confer a significant threat to the general population, hospitalized patients with critical illness and invasive lines, such as central venous catheters and enteral feeding tubes, have significant risk of morbidity and mortality if infected with *C. auris* [7,9]. Patients with comorbidities such as diabetes, recent surgery, or prior systemic antimicrobial use are also at increased risk of infection with *C. auris* [7,9]. While the presence of *C. auris* in the urinary tract, as with other Candida species, does not automatically indicate a UTI and could rather represent colonization, there is no specific criteria to aid in differentiating colonization from infection [1]. Critical, clinical decision making must be utilized in these at risk patient populations to determine if the isolation of *C. auris* in the urine necessitates antifungal treatment and should be based on the potential risks of not treating.

## 3. Identification/Susceptibilities

*C. auris* frequently goes undetected or is misidentified by common yeast-identifying, biochemical-based systems [12,13]. It is often incorrectly identified as *C. haemulonii, C. famata, C. sake*, and *C. parapsilosis* [7,12,13]. Cases of misidentification have been documented with the following systems: VITEK 2 YST, API 20C, BD Phoenix yeast identification system, and MicroScan [7]. The utilization of updated software or ‘libraries’ for these systems, the application of matrix-assisted laser desorption/ionization time-of-flight (MALDI-TOF), and molecular-based systems, such as real-time PCR assays, have made it possible to correctly identify *C.auris* [7]. Additional information on these identification technologies can be found on the Centers for Diseases Control and Prevention (CDC) website under “Identification of *Candida auris*” [7]. If these methods are unavailable at an institution within the United States, the CDC recommends that the location coordinate with the State Public Health Department to have the purported *C. haemulonii, C. famata, C. sake,* and *C. parapsilosis* isolates tested at a CDC lab [7].

The true susceptibility breakpoints of *C. auris* isolates in the serum are not specifically defined [7]. Tentative MIC breakpoints are provided by the CDC based on data from similar Candida species: ≥4 μg/mL, ≥2 μg/mL, and ≥32 μg/mL for anidulafungin/micafungin, amphotericin B/caspofungin, and fluconazole, respectively [7]. These estimates are provided based on serum MIC data, and do not take into consideration the concentration of antifungal in the urinary tract [23]. Lack of data to support MIC breakpoints for *C. auris,* particularly urinary isolates, further complicates antifungal treatment decision making for this often multi-drug resistant pathogen.

## 4. Review of Published Reports of *Candia auris* Isolates in Urine

Worldwide *C. auris* literature focuses largely on candidemia or other invasive infections, with very minimal data available on urinary isolation and other non-invasive sites and infections (respiratory tract, skin, etc.). However, brief reports on urine culture positivity rates as well as patient-specific culture data are found within this published information.

More generalized information on *C. auris* urinary culture positivity is available from a variety of countries, but epidemiology, pathology, susceptibility, and treatment are described for the entire study populations and are not provided based on urinary isolation subgroups. In a single center study conducted in Pakistan from 2014–2017, 193 *C. auris* strains were identified based on D1-D2 sequencing or by meeting pre-defined criteria [24]. Of the 193 strains, 83 were identified via urine cultures from indwelling catheters (73/83) or mid-stream samples (10/83) [24]. The 193 strains were collected from 92 individual patients, with 65 of those patients determined to have clinical infection and 19 diagnosed with a UTI without candidemia [24]. Antifungal therapy was deferred in 5/19 patients, 2 of these patients died within 24 h of the *C. auris* urine culture obtainment [24]. Two independent single-center European studies each reported urine culture positivity among *C. auris* infected populations [25,26]. There were 5 positive urine cultures isolated from 41 patients with candidemia in one study, with colonization preceding candidemia overall in 56% (23/41) of patients [25]. One potential urinary catheter infection (based on positive urine culture and signs and symptoms of infection requiring treatment) was identified among 50 infections in another study [26]. A report from Kuwait identified 314 *C. auris* isolates from 126 patients, of which 124 isolates were found in the urine [27]. Clinical *C. auris* infection was diagnosed in 49 patients in Russia from October 2016–December 2017 [28]. Positive culture sites included urine in 27 patients, urine/blood in 11 patients, blood/urine/tracheal aspirate in 5 patients, and urine/tracheal aspirate in 2 patients [28]. Reported cases of *C. auris* in New York were monitored from 2013–2017, with 51 clinical cases (defined based on intent of culture obtainment to diagnose or treat disease) identified with the initial site of culture positivity being the urine in 4 cases [29].

Published reports of *C. auris* in the urine provide inconsistent information, but 11 specific patient reports were found in the literature and are summarized in Table 1. Patient age was greater than 50 years in 70% (7/10) of cases, and males and females each made up 50% (4/8) of the population [8,30,31,32,33,34]. Age and gender were not reported in 1 and 3 cases, respectively. Additional sites of *C. auris* isolation were reported in 10 patients, with 80% (8/10) having at least two sites with *C. auris* identified on culture [8,30,31,32,33,34]. Concomitant candidemia was present in 60% (6/10) patients [8,30,31,32,33,34]. Among 11 individual patients, there were 12 *C. auris* isolates with susceptibility testing results provided (Table 2) [8,30,31,32,33,34]. Of note, isolates utilized for susceptibility testing may have been from a positive culture at an alternative site, and may not have been the first isolate collected. The results presented here may convey *C. auris* resistant strains that developed over time. Interpretation of results provided is based upon CDC recommendations for breakpoints as detailed previously. Fluconazole resistance was documented in 100% (12/12) of isolates collected [8,30,31,32,33,34]. Anidulafungin susceptibility was reported for 11 isolates, with 36% (4/11) being resistant [8,30,31,32,33,34]. Similarly, micafungin resistant isolates were noted in 42% (3/7) of reported cases [8,30,31,32,33,34]. Amphotericin B resistance rates were consistent with the echinocandins, with 42% (3/7) being resistant [8,30,31,32,33,34]. Antifungal treatment data was available for 10 patients, with 20% (2/10) of patients receiving no drug treatment [8,30,31,32,33,34]. Echinocandin therapy was reported in 88% (7/8) of cases receiving treatment [8,30,31,32,33,34]. Echinocandins were utilized as monotherapy in 57% (4/7) of cases, combination therapy with amphotericin B in 14% (1/7) of cases, part of an escalation/de-escalation stepwise therapy in 14% (1/7) of cases, and either as monotherapy or combination therapy in 14% (1/7) cases (information provided in prior publication is not specific) [8,30,31,32,33,34].

Persistence and mortality are frequently cited as factors associated with *C. auris* infections, and the data provided in the specific case reports of *C. auris* UTIs (Table 1) support these concerns. Patient 5′s cultures took 6 months to clear *C. auris* [31]. *C. auris* was isolated in Patient 10′s urine on day 7 and in the patient’s blood on day 22 [34]. Similarly, candiduria was identified on day 0 in Patient 11 and candidemia was identified on day 27 [8]. Patient 11′s urine and blood cultures remained positive on days 95 and 97, respectively [8]. Patients 7 and 8 only survived 3–4 weeks and 2 weeks, respectively, from the time of isolation of a pan-resistant *C. auris* culture [32].

## 5. Management of *C. auris* UTIs

### 5.1. Infection Control

Infection control measures are imperative in managing *C. auris* UTIs, as *C. auris* infections present risk of transmission similar to multi-drug resistant bacteria [7,35,36]. Proper use of personal protective equipment and handwashing or alcohol-based sanitizing is necessary to prevent patient-to-patient transmission by healthcare workers [7,35,36]. If possible, single patient rooms and designated equipment should be utilized in this patient population to prevent widespread transmission [7,35,36].

Appropriate cleaning practices are also key to the management of *C. auris* infection, as commonly utilized disinfectants are not guaranteed to be active against *C. auris* [7,37,38,39]. Specifically, agents with only quaternary ammonia compounds are not effective against *C. auris* [7]. The Environmental Protection Agency (EPA) has a list of registered, hospital-grade disinfectants with activity against *C. auris*, including agents with the following active ingredients: hydrogen peroxide, sodium hypochlorite, hydrogen peroxide/peroxyacetic acid, quaternary ammonium monomer + ethanol + isopropanol, hydrogen peroxide/octanoic acid/peroxyacetic acid [7,40]. Additionally, the EPA has approved exemptions to this list based on CDC evaluation of appropriate extra agents, with equivalent active ingredients as listed above [7,40]. Of note, instructions provided by the manufacturer on the exempted agents do not include specific guidance for *C. auris* disinfection, and instructions provided by the CDC (available online) should be followed [7].

### 5.2. Infection Surveillance

Surveillance screening aids in the prevention of *C. auris* transmission and should be utilized when either colonized or infected patients are identified [7,9]. After isolation of *C. auris* in the urine, contacts of the patient should be screened one time for colonization and potential subsequent infection. Screening of the healthcare environment should also be conducted in order to prevent, identify, and manage the potential for an outbreak within the institution. Surveillance should occur weekly within the patient environment and at discharge. If surveillance screening is positive after discharge, repeat screening should be conducted after cleaning to ensure appropriate disinfection was achieved.

### 5.3. Source Control

As with all other infections, source control is imperative as a first step in the management of individual patients with *C. auris* urinary colonization or UTIs. When possible, all invasive lines must be removed in order to prevent the transition from colonization to infection or remove the nidus of infection. A single center study from Pakistan detailed 11 UTIs that were catheter associated, and the catheter was removed as part of therapy in 8/11 patients [24]. In the same study, 17 catheterized patients were determined to be asymptomatically colonized in the urine, and the catheter was only removed in 10/17 patients [24]. These examples of incomplete adherence to source control practices highlight the need to consider source control as an integral part of the management of *C. auris* UTIs.

### 5.4. Antifungal Therapy

There is a paucity of data regarding the optimal treatment regimen for *C. auris* UTIs [7]. Commonly utilized and well-tolerated antifungals may not be adequate for the treatment of *C. auris* due to both intrinsic and developed resistance mechanisms [8,18,19,20,21]. Detailed here are specific considerations for each available class of antifungal agents, as well as a proposed treatment algorithm for *C. auris* UTIs.

#### 5.4.1. Triazole Antifungals

Triazole antifungals inhibit the synthesis of ergosterol, which is a main membrane sterol of fungi [41]. These agents are preferable for treatment given the availability of oral formulations [41]. Fluconazole is the drug of choice for most Candida UTIs, but the resistance patterns of *C. auris* (as discussed previously) make the triazole an inappropriate choice for empiric therapy. However, clinical decision making becomes more difficult when attempting to optimize therapy based on susceptibility reports. Prior case reports suggest that *C. auris* infections may persist after fluconazole therapy despite reported fluconazole susceptible isolates [8,33].

#### 5.4.2. Echinocandins

Echinocandin antifungals cause fungal cell lysis by inhibiting glucan synthesis [42]. These agents are extremely well tolerated compared to older antifungals such as amphotericin B and flucytosine, but are only available in intravenous formulations, which is less convenient compared to oral triazole options [42]. The significant resistance rates of *C. auris* strains to fluconazole, and presumptively the other triazoles, make echinocandins the first line agent for all *C. auris* infectious as they are predicted to be effective against 70% of *C. auris* isolates [7]. Although resistance rates of *C. auris* against the echinocandins are lower than those against the triazoles, resistance is still common and monotherapy may not be sufficient for the appropriate treatment of many UTIs [8,20,21].

#### 5.4.3. Amphotericin B

Amphotericin B causes cell lysis by binding to ergosterol and creating pore channels that alter the permeability of the fungal cell membrane and allow for leakage of monovalent ions [43]. Unlike the triazoles, amphotericin B also binds to mammalian cells, which leads to increased toxicity for the patient [43]. Less toxic, liposomal formations of the medication are available; however, these formulations do not concentrate well in the urine and are not considered appropriate for the treatment of any Candida UTIs based the IDSA guidelines for the treatment of Candidiasis [43,44]. Despite significant intolerance and toxicity associated with this agent, amphotericin B deoxycholate should be considered in the treatment of *C. auris* given the high resistance rates associated with better tolerated agents such as the triazoles and echinocandins [7,8,18,19,20,21,44].

#### 5.4.4. Flucytosine

Flucytosine is an antimetabolite that acts as an antifungal via the inhibition of DNA and protein synthesis [45]. Flucytosine is an older agent that is not commonly utilized due to potential for hepatic and hematological toxicities [45]. However, as with amphotericin B, the potential for triazole, echinocandin, and polyene resistant *C. auris* strains, necessitates the consideration of alternative agents in the management of *C. auris* UTIs. Flucytosine is an attractive alternative to the commonly utilized antifungal agents, as it is primarily excreted into the urine in its active form, and initial *C. auris* resistance to flucytosine is uncommon based on current knowledge [22,45]. The utilization of flucytosine in multi-drug resistant strains of *C. auris* is consistent with current guideline recommendations for the treatment of another antifungal resistant Candida strain, *C. glabrata* [44]. Unfortunately, the development of candidal resistance to flucytosine during the course of therapy has been documented with other species, and it is reasonable to assume that this could also occur with *C. auris*, further highlighting the importance of repeat susceptibility testing throughout the course of drug therapy [44,46]. The potential for acquired resistance makes monotherapy with flucytosine an inappropriate treatment option for *C. auris* UTIs. An additional challenge encountered with flucytosine therapy is the lack of availability across the world. Per a 2016 estimate, flucytosine was not available in 78.3% of countries, but where available and accessible it should be considered for use in treating *C. auris* UTIs [47].

#### 5.4.5. Combination Therapy

Despite lack of evidence, combination therapy has been utilized in multiple case reports, including an echinocandin + amphotericin B, a triazole + an echinocandin, or flucytosine + amphotericin B [8,30,31,32,33,34,48].

Among the specific cases with antifungal therapy reported in Table 1, 50% (4/8) of patients were treated with combination therapy [8,30,31,32,33,34]. Based on the significant mortality and the antifungal resistance rates associated with *C. auris* infections, combination therapy may be a highly effective treatment strategy for persistent, recurrent, and resistant infections.

#### 5.4.6. Amphotericin B Bladder Irrigations

The utilization of conventional amphotericin B bladder irrigations dates back to the 1960s and is theoretically useful for the treatment of lower UTIs [49,50,51]. The practice is controversial due to limited efficacy data and the availability of well-tolerated systemic antifungal agents, as the initial impetus to use amphotericin B bladder irrigations was to avoid the toxicity associated with older systemic antifungals [49]. However, the potential inactivity of antifungal agents against *C. auris* may necessitate the use of alternative treatment options. If a *C. auris* infection is persistent despite the exhaustion of systemic options, direct installation of amphotericin B may be an alternative option to control the infection. While this method will not be utilized for treatment in the majority of *C. auris* UTI patients, it should be considered within the treatment algorithm for persistent, recurrent, and resistant infections.

### 5.5. Treatment Algorithm

**Figure 1:**
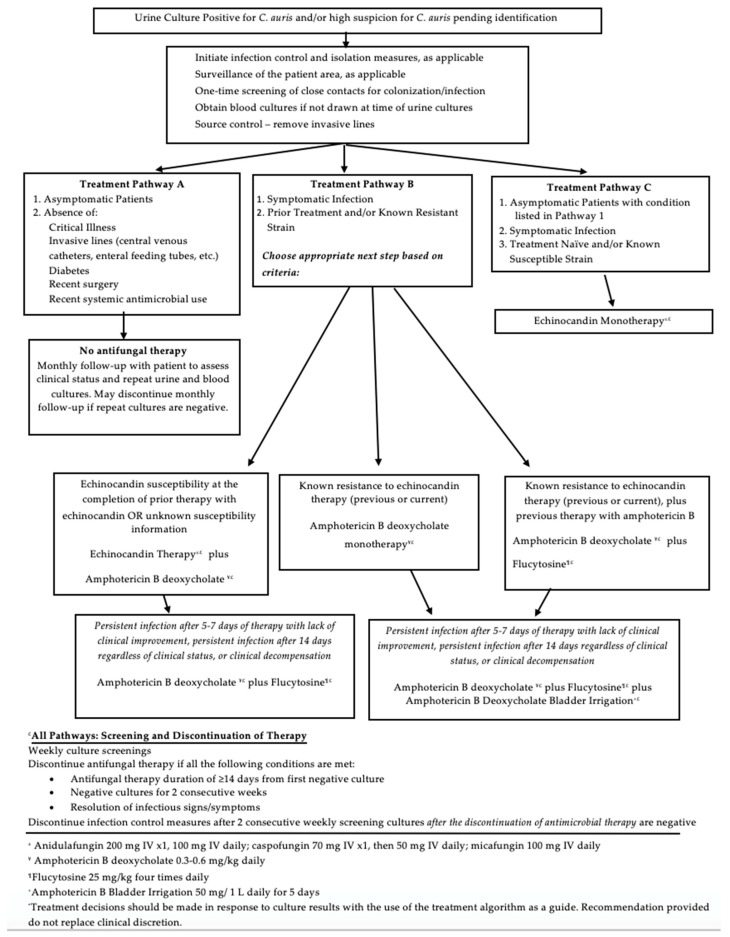
Treatment algorithm for *Candida auris* UTIs.

Figure 1 details a proposed treatment algorithm for *C. auris* UTIs. The algorithm is based on patient clinical status, the presence or absence of clinical infection, comorbidities, infection, and therapy history. Appropriate infection and source control measures, as delineated above, should be utilized in all patients with *C. auris* identified on culture. The CDC recommends against the antifungal treatment of *C. auris* isolated from non-invasive sites such as the urine [7]. In the proposed guidance, we recommend against treatment only if the patient meets specific criteria, as is detailed in Treatment Pathway A. In Treatment Pathway C, monotherapy with an echinocandin is the first line treatment for patients meeting clinical, microbiological, and treatment history criteria, which is consistent with CDC recommendations for invasive *C. auris* infections [7]. Treatment Pathway B provides decision making support for more difficult infections, and echinocandin monotherapy is not considered appropriate for patients meeting criteria for this treatment pathway. Treatment decisions should be based on prior therapy, and prior and current culture susceptibility results (if available), as detailed in the algorithm. Therapy alterations in response to persistent and recurrent infections should be based on prior therapy, repeat culture susceptibility data, and clinical status of the patient. Repeat cultures throughout therapy are recommended due to concern over the development of resistance during active antifungal therapy. Bladder irrigation in addition to systemic antifungal therapy should be considered if the infection persists despite combination systemic therapy. Even in patients with concomitant candidemia, bladder irrigation should still be considered given the potential for the urinary infection to be the source of the candidemia.

### 5.6. Eradication Screening

Repeated screening of infected or colonized patients during and after therapy should be utilized in order to ensure the efficacy of therapy and prevent the spread of *C. auris* [7,9]. While end of treatment cultures are not common with most infections, *C. auris* is an unfamiliar pathogen that is associated with persistent infection and significant mortality. Test of eradication is the most effective method to identifying treatment failures and persistent infection in order to guide if continued therapy is necessary and to prevent the spread of infection. Cultures should be obtained weekly from previously positive sites as well as the blood until criteria for discontinuation of infection control measure are met, as detailed below. Antifungal therapy may be discontinued when culture negativity is documented on two consecutive weekly screenings, as long as all clinical signs and symptoms of infection have resolved. Antifungal therapy should be continued for a minimum of 14 days after first negative culture, as is recommended by Infectious Diseases Society of America (IDSA) for the treatment of candidemia [44]. Adherence to this guidance by the IDSA was chosen based on the mortality associated with *C. auris* infections and a lack of data to guide duration of therapy specifically for *C. auris* infections. Additional screening of the axilla and groin can be considered if an institution feels that it would be of clinical utility, this practice is currently supported by the CDC [7]. Once antifungal therapy is discontinued, weekly cultures should be continued to guide the discontinuation of infection control measures. Two negative cultures, one week apart, are adequate to consider the discontinuation of infection control measures. However, it is important to consider the potential for *C. auris* to remain undetected even if present, and the impact that may have on false negative screening of previously colonized or infected patients. Patients previously colonized or infected with *C. auris* may have repeated, negative cultures, and then subsequent positive cultures. While this could potentially represent new infection with *C. auris*, given the commonality of this pathogen going undetected, and lack of truly effective treatment regimens, it is also possible that this could indicate persistent infection. The official discontinuation of infection control precautions should be determined on a case-by-case basis, with the awareness that colonization or infection may still be present. Patient follow-up should be conducted one month after the discontinuation of infection control measures, including repeat cultures.

## 6. Discussion

*Candida auris* is a novel, unfamiliar pathogen that has quickly become of worldwide concern since its discovery in Japan in 2009 [10]. Multiple factors play a role in making *C. auris* a pathogen of concern, starting with the need for updated identification systems and software (MALDI-TOF, PCR assays, etc.) to prevent the misidentification of *C. auris* as other Candida species, such as *C. haemulonii*, *C. famata*, *C. sake*, and *C. parapsilosis* [8,10,11,12,13]. After proper identification, enhanced source control measures are required to stop the spread of *C. auris*, as it persists on surfaces and in the environment in a fashion similar to MDR bacteria [7]. *C. auris* infections are also often prolonged, presenting challenges in both determining durations of therapy as well as deciphering colonization from persistent infection, and further, from recurrent infection [7,8,17]. Perhaps the ultimate challenge that *C. auris* presents is the significant resistance patterns the isolates commonly express, limiting the availability of efficacious antifungal treatment options [7,18,19,20,21].

Candida species are frequently isolated in the urine, however details of *C. auris* in the urinary tract are not well documented in the literature that is published on the pathogen. What is apparent throughout the available literature, however, is that urine isolates comprise a significant portion of *C. auris* cultures found within surveillance study populations, and that there is a vast uncertainty of how to properly manage *C. auris* urinary tract infections once diagnosed [8,24,25,26,27,28,29,30,31,32,33,34]. In one study, *C. auris* was isolated in the urine in 92% of patients reviewed, demonstrating that the urine is likely a key site in *C. auris* infections [28]. The treatment of patients with *C. auris* in the urine has historically been highly variable and ranged from no treatment to combination antifungal therapy for prolonged durations. Even source control, a quintessential component of all infection management, was not conducted in 36% of patients with *C. auris* isolated in the urine in a published report [24]. Among the 11 specific case reports we identified (Table 1), concomitant candidemia was reported in 60% of patients, highlighting the potential for *C. auris* urinary isolation to serve as early indication of invasive infection and the importance of obtaining blood cultures at the time of urinary isolation [8,30,31,32,33,34]. Two patient cases reporting the isolation of a pan-resistant *C. auris* culture documented a time to patient death of 2–4 weeks, which supports the notion that these infections should be identified early and managed aggressively [32].

The current body of literature does not provide any consistent evidence for a preferred management strategy for patients with *C. auris* UTIs. Although the CDC recommends against treating non-invasive *C. auris* infections, we believe that the risk for persistent, pan-resistant infection and potential for morbidity and mortality that is apparent throughout published reports necessitates treatment of most *C. auris* infections, regardless of isolation site [7]. Deferring treatment at the time of urinary isolation may put the patient at increased risk of invasive infection, at which point *C. auris* is incredibly difficult to treat effectively. Despite lack of evidence to guide the optimal utilization of antifungal agent(s) for *C. auris* infections, we recommend a systematic approach (Figure 1) to therapy selection based on currently known resistance data. Echinocandins and amphotericin B deoxycholate present the most promising data for efficacy against *C. auris* isolates [7,8,20,21,22]. For this reason, our treatment pathways begin with monotherapy with either of these agents, or, more often, combination therapy. However, current data suggests that echinocandin and polyene resistance is common among *C. auris* isolates, and treatment failure remains a concern [7,8,20,21,22]. Therefore, it is imperative for the treatment algorithm to include additional antifungal agent considerations, such as flucytosine and amphotericin B deoxycholate bladder irrigations.

Triazole antifungals were left out of the proposed treatment algorithm. Based on available literature, *C. auris* resistance rates to fluconazole approach 100%, and there is a lack of data regarding resistance rates to the other triazole agents [7,8,18,19,30,31,32,34]. Until more data on the utilization of these agents becomes available, we are not comfortable recommending these agents for the treatment of *C. auris*, with a preference to use agents that have a higher likelihood of being effective, such as echinocandins and amphotericin B.

Novel agents for the treatment of *C. auris* are needed. Potential agents for investigation include ibrexafungerp, nitroxoline, and the rocaglates class [52,53,54].

## 7. Conclusions

Robust data to support any therapy recommendations for the treatment of *C. auris* UTIs is not available. However, these infections are growing in prevalence and represent a large burden to both the healthcare system and patients. The treatment algorithm proposed here is a culmination of the currently available data. The approach focuses on the aggressive treatment of *C. auris* in most patients to hopefully prevent subsequent invasive spread, multi-drug resistance, and ultimately mortality. Moving forward, it is imperative that information regarding *C. auris* urinary isolation, concomitantly positive sites, treatment management, including both infection control measures and antifungal agent selection, and duration of therapy is collected and disseminated in order to design evidence based, effective treatment regimens for patients infected with this unique Candida species. The treatment approach to these infections will continue to evolve as more data becomes available.

## Figures and Tables

**Table 1:** Review of published reports of *Candia auris* isolates in urine ^1^.

Patient	Year	Age (y), Sex	Medical History ^2^	Site	Prior Antifungal Treatment	Treatment ^3^	Infection Diagnosis	Clinical Outcome
1 [30]	2016–2017	62, M	Current: central venous catheter, ICU admission PMH: cerebrovascular accident, obstructive uropathy	Blood Urine	Yes	AFG	Candidemia	Survived to day 30
2 [30]	2016–2017	31, F	Current: central venous catheter, ICU admission PMH: kidney transplant, systemic lupus erythematosus, *Pneumocystis jirovecii* pneumonia, Cytomegalovirus infection, pneumonitis	Blood Urine	Yes	AFG	Candidemia	Deceased
3 [31]	2017	67, F	PMH: intracranial hemorrhage	Urinary catheter Groin Trach	N/A	None	None	Transferred to rehabilitation facility
4 [31]	2017	48, F	Current: neurologic disorder	Urinary catheter	N/A	None	None	Discharged alive
5 [31]	2017	15, M	Not available	Urine Blood	N/A	LipAmpB VRC	Candidemia	Cultures–6 months to clear
6 [31]	2017	60, M	Not available	Urine	N/A	N/A	None	N/A
7 [32]	2018	>50, N/A	Current: chronic mechanical ventilation, alcohol dependence	Urine Tracheal aspirate	No	Echinocandin	Noted to have infection	Deceased
8 [32]	2017	>50, N/A	Current: chronic ventilator dependence	CVC tip Blood Urine	No	Echinocandin AmpB	Candidemia	Deceased
9 [33]	2016	N/A	Current: paraplegia with long-term, indwelling Foley catheter	N/A	N/A	Fluconazole	Not stated	Survived
10 [34]	2017	70, F	Current: congestive cardiac failure, cellulitis, acute kidney injury on hemodialysis, brain infarcts, duodenal ulcer, H. pylori infection PMH: heart failure, ischemic heart disease, chronic kidney disease, diabetes, hypertension	Urine Blood	No	AFG	Candidemia Urosepsis	Deceased
11 [8]	2018	54, M	Current: sepsis, DVT, tracheostomy, enteric feeding tube, colostomy PMH: quadriplegia, chronic wounds, osteomyelitis with abscess	Urine Blood	Yes	MFG POS FLU	Candidemia	Discharged on long-term antifungal and antimicrobial therapy

^1^ N/A: information not available from source literature; ^2^ ICU: intensive care unit; PMH: past medical history; ^3^ AFG: anidulafungin; LipAmpB: liposomal amphotericin B; VRC: voriconazole; MFG: micafingin; POS: posaconazole; FLU: fluconazole.

**Table 2:** Antifungal susceptibility data of *C. auris* isolates from patient cases in Table 1.

Patient	Susceptibility Testing Method	Minimum Inhibitory Concentrations ^1^									
		FLU ^2^	ITR ^3^	ISA ^4^	POS ^5^	VRC ^6^	CAS ^7^	MFG ^8^	AFG ^9^	AmpB ^10^	Flucy ^11^
1	YestOne Sensititre	128	0.12	N/A	0.06	0.5	0.12	0.12	0.12	1	8
2	YestOne Sensititre	256	0.25	N/A	0.12	2	0.12	0.12	0.12	2	0.12
3	Broth Microdilution	≥64	2	1	0.5	2	N/A	N/A	0.25	N/A	N/A
4	Broth Microdilution	≥64	0.25	≤0.016	≤0.016	0.25	N/A	N/A	0.125	N/A	N/A
5	Broth Microdilution	≥64	0.5	0.25	≤0.016	0.5	N/A	N/A	16	N/A	N/A
6	Broth Microdilution	≥64	0.25	≤0.016	0.06	2	N/A	N/A	0.5	N/A	N/A
7	Broth Microdilution/E-Test	>256	N/A	N/A	0.5	2	2	4	4	2	N/A
8	Broth Microdilution/E-Test	>256	N/A	N/A	0.25	2	16	4	4	2	N/A
9	N/A	N/A	N/A	N/A	N/A	N/A	N/A	N/A	N/A	N/A	N/A
10	Broth Microdilution	64	0.031	<0.016	<0.016	0.125	N/A	0.063	0.031	2	N/A
11	YestOne Sensititre/Broth Microdilution	2	N/A	≤0.03	N/A	0.015	0.06	0.12	0.12	1	N/A
		2	N/A	≤0.03	N/A	0.06	>8	>8	4		

^1^ N/A: information not available from source literature; ^2^ FLU: fluconazole; ^3^ ITR: itraconazole; ^4^ ISA: isavuconazole; ^5^ POS: posaconazole; ^6^ VRC: voriconazole; ^7^ CAS: caspofungin; ^8^ MFG: micafungin; ^9^ AFG: anidulafungin; ^10^ AmpB: amphotericin B; ^11^ Flucy: flucytosine.
